# Hydrogen Sulfide-to-Thiosulfate Ratio Associated with Blood Pressure Abnormalities in Pediatric CKD

**DOI:** 10.3390/jpm12081241

**Published:** 2022-07-29

**Authors:** Chien-Ning Hsu, Wei-Ling Chen, Wei-Ting Liao, Guo-Ping Chang-Chien, Sufan Lin, You-Lin Tain

**Affiliations:** 1 Department of Pharmacy, Kaohsiung Chang Gung Memorial Hospital, Kaohsiung 833, Taiwan, China; cnhsu@cgmh.org.tw; 2 School of Pharmacy, Kaohsiung Medical University, Kaohsiung 807, Taiwan, China; 3 Department of Pediatrics, Kaohsiung Chang Gung Memorial Hospital, Kaohsiung 833, Taiwan, China; weilingchen@cgmh.org.tw (W.-L.C.); weiting0307@cgmh.org.tw (W.-T.L.); 4 Center for Environmental Toxin and Emerging-Contaminant Research, Cheng Shiu University, Kaohsiung 833, Taiwan, China; guoping@csu.edu.tw (G.-P.C.-C.); linsufan2003@csu.edu.tw (S.L.); 5 Super Micro Mass Research and Technology Center, Cheng Shiu University, Kaohsiung 833, Taiwan, China; 6 Institute of Environmental Toxin and Emerging-Contaminant, Cheng Shiu University, Kaohsiung 833, Taiwan, China; 7 College of Medicine, Chang Gung University, Taoyuan 330, Taiwan, China

**Keywords:** cardiovascular disease, ambulatory blood pressure monitoring, chronic kidney disease, children, hydrogen sulfide, nitric oxide, hypertension, thiosulfate

## Abstract

Identifying children with chronic kidney disease (CKD) at high risk of cardiovascular disease (CVD) and ensuring they receive appropriate treatment can prevent CVD events and mortality later in life. Hydrogen sulfide (H_2_S) is a gaseous signaling molecule participating in CVD and CKD. Thiosulfate is not only an oxidation product of H_2_S but is also a H_2_S donor. We examined whether H_2_S, thiosulfate, and their combined ratio have differential associations with CVD risk markers in 56 children and adolescents aged 6–18 years with CKD stages G1–G4. Up to two-thirds of CKD children showed higher BP load on 24 h ambulatory blood pressure monitoring (ABPM), even in the early stage. CKD children with ABPM abnormalities had a higher H_2_S-to-thiosulfate ratio, while H_2_S-related parameters were not affected by the severity of CKD. The H_2_S-to-thiosulfate ratio was positively correlated with 24 h systolic BP (SBP), nighttime SBP, and carotid artery intima-media thickness (cIMT). After adjusting for confounders, H_2_S was negatively associated with LV mass, thiosulfate was positively associated with 24-DBP, and the H_2_S-to-thiosulfate ratio was positively correlated with nighttime SBP and cIMT. Our data demonstrate differential associations in circulating H_2_S, thiosulfate, and their combined ratio with CVD risk in childhood CKD. Further studies are required to determine whether targeting the H_2_S signaling pathway can develop novel therapeutic strategies against CVD in this high-risk population.

## 1. Introduction

Hydrogen sulfide (H_2_S) is a gaseous signaling molecule of biological impact in illness involving cardiovascular disease (CVD) [1,2]. Despite being a poisonous gas in excess, H_2_S executes important biological functions at the physiological level [3]. Nitric oxide (NO) has long been known as a vasodilator. Increasing experimental evidence indicates that NO deficiency is involved in hypertension and chronic kidney disease (CKD) [4]. Similar to NO, H_2_S has a key role in the regulation of blood pressure (BP) and renal physiology [1,5]. The production of H_2_S can occur through enzymatic or non-enzymatic reactions [1]. H_2_S can be non-enzymatically generated from organic thiol. Thiosulfate is one product formed during oxidative H_2_S metabolism. Alternatively, thiosulfate can be reduced and regenerate H_2_S [6]. Accordingly, thiosulfate is a metabolite of H_2_S and an index of the sulfide pool [7]. In adult patients who received hemodialysis, the H_2_S-generating pathway was down-regulated [8].

Hypertension in children and adults with CKD is an important clinical concern resulting in high risk of CVD morbidity and mortality as well as CKD progression. Although overt cardiovascular (CV) events rarely happen in children, atherosclerosis and hypertension can begin in early childhood [9]. We and others have shown that hypertension is extraordinarily prevalent in CKD children, even in the early stages [10,11]. Accordingly, early identification of children at risk for CVD may facilitate timely intervention and reduce the burden of CKD. To date, several noninvasive functional and structural assessments have been reported to identify high-risk CKD children for CVD [12]. These surrogate markers for CVD include 24 h ambulatory blood pressure monitoring (ABPM), carotid artery intima-media thickness (cIMT), left ventricular (LV) mass, and arterial stiffness indices. [12,13,14]. Our previous study revealed that several NO-related parameters are correlated with certain CVD risk surrogate markers in the early stages of pediatric CKD [10]. However, little information currently exists with regard to H_2_S and thiosulfate levels in CKD children [15]. Importantly, early identification of subphenotypes has led to insights into their pathogenesis and the development of personalized approaches to CKD care.

Given this background, we hypothesized that the H_2_S signaling pathway may be a crucial mechanism contributing to the development of hypertension and CVD in childhood CKD. We therefore evaluated the associations between H_2_S, thiosulfate, and their combined ratio with CVD risk markers in children with early-stage CKD.

## 2. Materials and Methods

### 2.1. Patients and Study Design

We used data from a prospective cohort study of children and adolescents aged 6–18 years with CKD who were recruited between November 2018 and April 2022 from the Pediatric Nephrology Outpatient Clinic at the Kaohsiung Chang Gung Memorial Hospital, a tertiary medical center in Taiwan. This project was approved by the ethics committee of Chang Gung Medical Foundation, Taoyuan, Taiwan (201701735A3C501 and 202001973A3C601), and written informed consent was obtained from each participant and their parents. The CKD was defined and staged by the KDIGO 2012 clinical practice guidelines [16]. Based on body height and blood creatinine (Cr) level, the estimated glomerular filtration rate (eGFR) was calculated with the bedside CKiD equation formula [17]. Staging of CKD was classified according to eGFR (mL/min/1.73 m^2^) as G5 < 15, G4 15–29, G3 30–59, G2 60–89, and G1 ≥ 90. The etiologies of CKD were divided into two groups: congenital anomalies of kidney and urinary tract (CAKUT) and non-CAKUT. The following exclusion criteria were applied: if a participant was pregnant, had congenital heart disease, had CKD stage G5, had received dialysis or a kidney transplant, or was unable to cooperate with the CV assessment, then they were excluded from the selection.

This ancillary study was conducted on a subgroup of 56 participants who received the measurement of H_2_S and thiosulfate, 24 h ABPM, as well as CV assessments. We collected fasting blood samples and spot urine samples. These aliquots were refrigerated immediately before being moved to −80 °C storage. Blood urea nitrogen, Cr, hemoglobin, total cholesterol, triglyceride, low-density lipoprotein (LDL), glucose, sodium, potassium, calcium, phosphate, uric acid, and total protein-to-creatinine ratio were analyzed by the hospital central laboratory as stated before [11].

### 2.2. Analysis of Plasma H_2_S and Thiosulfate Levels

We measured concentrations of H_2_S and thiosulfate in the plasma according to our validated protocol by an Agilent Technologies 1290 high-performance liquid chromatography (HPLC) system connected to an Agilent 6470 Triple Quadrupole LC/Mass Spectrometry (MS) (Agilent Technologies, Wilmington, NC, USA) [18]. Phenyl 4-hydroxybenzoate (PHB) was added to samples as an internal standard. The column used for chromatographic separation was a Supelco C18 column (5 cm × 2.1 mm, 3 µm; Sigma–Aldrich, Bellefonte, PA, USA) protected by an Ascentis C18 column (2 cm × 2.1 mm, 3 µm; Merck KGaA, Darmstadt, Germany). The solvent system was composed of 0.1% formic acid in water and acetonitrile. The flow rate was 300 µL/min. The LC/MS was equipped with an electrospray ionization (ESI) source. We detected thiosulfate derivative pentafluorobenzyl (PFB)-S_2_O_3_H and H_2_S derivative sulfide dibimane (SDB). Selected reaction monitoring mode was applied to detect target compounds with a target of *m*/*z* 415–223, *m*/*z* 292.99–81, and *m*/*z* 212.99–93, for SDB, PFB-S_2_O_3_H, and PHB, respectively. The intra-assay coefficient of variation was 4% and 6% for H_2_S and thiosulfate, respectively.

### 2.3. Blood Pressure Measurement and Cardiovascular Assessment

Children were instructed to measure office BP at the clinic visit. After an initial 5 min of rest, seated BP was measured in triplicate at 1 min intervals. Hypertension was diagnosed according to the 2017 American Academy of Pediatrics (AAP) guidelines [19]. Data from 24 h ABPM were collected from subjects using the Oscar II monitoring device (SunTech Medical, Morrisville, NY, USA). BP and heart rate (HR) were recorded by 24 h ABPM every 20–30 min for 24 h. Participants and their parents were asked to keep a personal record documenting activities and sleeping times. We used the following definition to examine abnormalities on ABPM profile: (1) daytime or nighttime systolic or diastolic BPs greater than the 95th percentile in reference to gender and height; (2) daytime or nighttime systolic or diastolic BP load greater than 25%; and (3) nighttime BP load dipping less than 10% based on comparison with ABPM reference data [20].

Echocardiograms were performed on Philips IE33 system (Philips, Bothell, WA, USA) by experienced pediatric cardiologists. For each patient, measurement of LV mass was performed in the parasternal long-axis view using two different modalities (M-mode imaging and 2D imaging). The LV mass index (LVMI) was calculated by indexing LV mass to height^2.7^ [21]. Images of the common carotid artery were obtained using Doppler ultrasound (ProSound α7, Aloka Co., Tokyo, Japan) with a 5–12 MHz linear array transducer. The cIMT was measured during end diastole as determined at the R-wave on the electrocardiography. Indices of arterial stiffness, augmentation index (AI), and pulse wave velocity (PWV) were analyzed by echo-tracking technique (e-TRACKING system; Aloka Co., Tokyo, Japan).

### 2.4. Statistical Analysis

Descriptive characteristics were expressed as median (25–75th percentile), mean ± standard deviation, or number (%). Mann–Whitney *U*-test, *t*-test, or Chi-squared test was employed to evaluate differences in variables between the two groups. Spearman’s rank correlation coefficient analysis was performed to measure the degree of association between two variables. Multivariable linear regression models were used to examine the associations among H_2_S-related parameters with CVD risk markers. The level of statistical significance was set at *p* < 0.05. All data were analyzed using the Statistical Package for the Social Sciences (SPSS) software 14.0 (Chicago, IL, USA).

## 3. Results

### 3.1. Patient Characteristics

As Table 1 shows, the study participants comprised 56 children and adolescents with CKD. This study group had a median age of 11.4 years, was 57.1% male, and consisted of 51.8% with CAKUT. Among the 56 subjects with CKD stages G1–G4, 35 (62.5%), 17 (30%), 3 (5.4%), and 1 (1.8%) had CKD stages G1, G2, G3, and G4, respectively. CKD children and adolescents were stratified into two groups based on eGFR: the G1 group (eGFR ≥ 90 mL/min/1.73 m^2^) and the G2–G4 group (eGFR < 90 mL/min/1.73 m^2^). The G2–G4 group was higher in age, systolic BP, blood urea nitrogen, Cr, and uric acid, but lower in eGFR than those in the G1 group (Table 1). A total of 12 CKD children (21.4%) were diagnosed as having hypertension according to office BP measurement; however, CKD children with this characteristic did not differ between the two groups. Regarding antihypertensive therapy, five of the G1 group (14.3%) and six of the G2–G4 group (28.6%) received angiotensin II receptor blocker (ARB) alone or with calcium channel blocker.

### 3.2. Plasma H_2_S and Thiosulfate Levels

**Figure 1:**
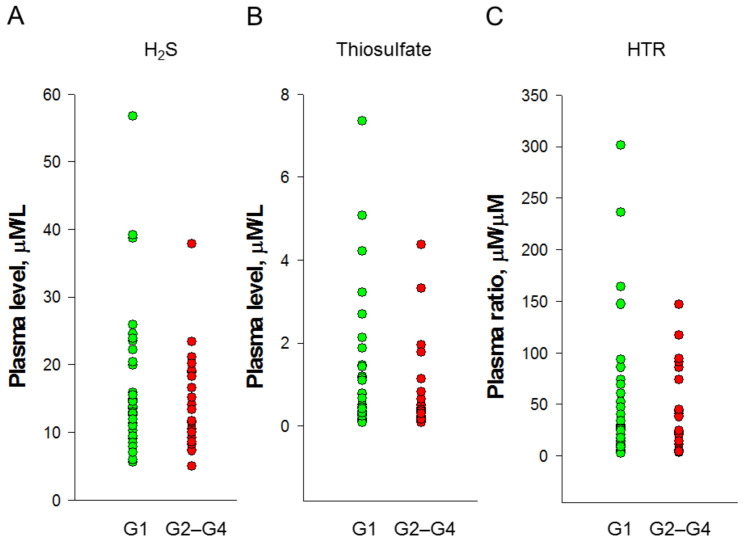
Plasma (**A**) H_2_S and (**B**) thiosulfate levels and (**C**) H_2_S-to-thiosulfate ratio (HTR) in CKD children.

We analyzed the plasma levels of H_2_S and thiosulfate in children with CKD and determined the ratio of H_2_S to thiosulfate (Table S1, Supplementary Materials). As presented in Figure 1, our data revealed there was no difference in plasma levels of H_2_S (Figure 1A), thiosulfate (Figure 1B), nor in the ratio of H_2_S to thiosulfate (Figure 1C) between the two groups. Analyses that evaluated the existence of associations between levels of H_2_S-related parameters with renal function and office BP indicated that there was no correlation between plasma Cr level with H_2_S (*p* = 0.565), thiosulfate (*p* = 0.408), nor the ratio of H_2_S to thiosulfate (*p* = 0.448). Additionally, systolic BP was positively correlated with plasma H_2_S level (*r* = 0.264, *p* = 0.049).

### 3.3. Cardiovascular Assessment

From the study of 56 pediatric patients with CKD who concurrently had laboratory tests and a comprehensive CV assessment performed, 38 (67.9%) had at least one BP load abnormality on ABPM. This included 11 subjects (19.6%) with 24 h BP greater than the 95th percentile, 6 subjects (10.7%) with daytime BP greater than the 95th percentile, 18 subjects (32.1%) with nighttime BP greater than the 95th percentile, 25 subjects (44.6%) with BP load greater than the 25th percentile, and 33 patients (58.9%) with a nocturnal decrease in BP of less than 10% (Table 2). There was a greater proportion of CKD children with 24 h BP greater than the 95th percentile in the G2–G4 group vs. the G1 group. We observed that the LV mass was higher in children with CKD stages G2–G4 compared to G1, while LVMI did not differ between the two groups. Likewise, the cIMT in the G1 and G2–G4 group did not differ. Using the AI and PWV to evaluate arterial stiffness, we observed neither was different between the two groups.

### 3.4. Association between H_2_S and Cardiovascular Risk Markers

Table 3 illustrates correlations between H_2_S, thiosulfate, and H_2_S-to-thiosulfate ratio with CV risk markers across all CKD patients. Spearman’s rank correlation analysis revealed H_2_S was positively correlated with nighttime SBP (*r* = 0.275, *p* = 0.04). Conversely, thiosulfate negatively correlated with nighttime SBP (*r* = −0.27, *p* = 0.044). The H_2_S-to-thiosulfate ratio exhibited positive correlations with 24 h SBP (*r* = 0.306, *p* = 0.002), nighttime SBP (*r* = 0.336, *p* = 0.011), and cIMT (*r* = 0.267, *p* = 0.047).

We next analyzed plasma H_2_S and thiosulfate levels, and their combined ratio, stratified according to the ABPM profile. Table 4 shows the H_2_S-to-thiosulfate ratio was significantly higher in CKD children with 24 h hypertension, nighttime hypertension, high BP load, non-night dipping, and abnormal ABPM profile. Analysis of individual H_2_S or thiosulfate did not show any significant difference between CKD children with normal and abnormal ABPM profile.

Table 5 illustrates the associations between H_2_S, thiosulfate, and their ratio with cardiovascular risk markers using multivariate linear regression analyses adjusted for age, sex, eGFR, uric acid, and H_2_S metabolites. In applications of regression analysis for prediction models (*r* = 0.488, *p* = 0.002), night SBP was associated with the H_2_S-to-thiosulfate ratio (*p* = 0.009). Additionally, thiosulfate was associated with 24 h DBP (*p* = 0.004), daytime DBP (*p* = 0.012), and nighttime DBP (*p* = 0.012), controlling for age and eGFR. We also noted that LV mass was negatively associated with H_2_S (*p* = 0.004) in the adjusted model controlling for age (*r* = 0.765, *p* < 0.001). Moreover, a positive association was found between cIMT and the H_2_S-to-thiosulfate ratio (*p* = 0.021) in the adjusted model controlling for sex (*r* = 0.458, *p* = 0.004). Our data suggest a significant influence of the H_2_S pathway on the CVD risk.

## 4. Discussion

This is the first study describing the different predictive abilities of plasma H_2_S, thiosulfate, and H_2_S-to-thiosulfate ratio as biomarkers for subclinical CVD in CKD children. The key findings are (1) H_2_S-related parameters were not affected by the severity of CKD; (2) more than two-thirds of CKD children exhibited BP load abnormalities on ABPM, even in the early stage; (3) the H_2_S-to-thiosulfate ratio was positively correlated with 24 h SBP, nighttime SBP, and cIMT; (4) CKD children with abnormalities on ABPM had a higher H_2_S-to-thiosulfate ratio compared to those with a normal profile; and (5) linear regression models indicated H_2_S was inversely associated with LV mass; thiosulfate was positively associated with 24-DBP and daytime and nighttime DBP; and the H_2_S-to-thiosulfate ratio was positively correlated with nighttime SBP and cIMT.

Plasma H_2_S level has been reported to be reduced in CKD patients and animal models [5,8,18], possibly due to inhibition of H_2_S-generating enzyme expression by CKD. Conversely, sodium thiosulfate therapy aids in preventing CKD-induced hypertension accompanied by increases in plasma levels of H_2_S and thiosulfate [18]. Notably, thiosulfate can serve as a sulfide donor to increase H_2_S. In contrast, a significant quantity of H_2_S is oxidized to thiosulfate. Accordingly, we determined not only H_2_S and thiosulfate levels but also calculated their combined ratio to investigate their bidirectional relationship. The ratio of H_2_S-to-thiosulfate may reflect the recycling of H_2_S [22].

Plasma H_2_S and its metabolites have served as biomarkers for many diseases involving CVD [23]. So far, a plethora of different analytical methods has evolved for H_2_S detection. Given the diverse chemistries of H_2_S detection methods, orders of magnitude differences in the physiological sulfide levels have been reported with a relatively high concentration [23]. Our data analyzed by the HPLC-MS/MS method tie well with recently developed methods revealing that the free H_2_S level in plasma is relatively low, as reported at low µmol/L level [24,25]. Importantly, in addition to its free forms, within the biological matrix, H_2_S also presents in other bound forms, which are involved in releasing free H_2_S in physiological response to stimulus [23,24].

We observed that there was no difference of plasma levels of H_2_S, thiosulfate, and H_2_S-to-thiosulfate ratio in CKD children between the G1 and the G2–G4 groups. These results contradict previous observations in adult patients with CKD, in whom plasma H_2_S levels gradually decreased with CKD stage climbing [25]. We believe that the population comprising this study differs somewhat from those formerly reported since a significant fraction of children were in the early stages of CKD. 

Our study shows that a high prevalence of CKD children had BP load abnormalities on ABPM irrespective of their CKD stages. Our data indicated that high BP load on ABPM in children with early stages of CKD is often disguised by office BP measurements, consistent with prior studies [10,11]. In line with prior findings in pediatric CKD [21,26], left ventricular hypertrophy represented as increased LV mass tends to develop in advanced CKD. However, several CVD risk markers, such as cIMT, AI, and PWV, were not different between the two groups.

Although there were few associations between abnormal ABPM profile with individual H_2_S or thiosulfate level, the H_2_S-to-thiosulfate ratio was significantly higher in CKD children with BP load abnormalities on ABPM, including 24 h hypertension, nighttime hypertension, high BP load, and non-night dipping. Additionally, in our study, a positive association between the H_2_S-to-thiosulfate ratio and nighttime SBP was found, even after controlling for confounding. We observed that there were strong positive associations between thiosulfate with 24 h DBP, daytime DBP, and nighttime DBP. These findings are in remarkable contrast to recent animal studies showing thiosulfate had therapeutic potential in hypertension and kidney disease [18,22,27]. Considering that H_2_S and thiosulfate can be converted to each other, a high H_2_S-to-thiosulfate ratio may reflect increased release of free H_2_S or reduced sulfide pool capacity to augment the thiosulfate-to-H_2_S conversion. This mechanistic relationship supports the notion that the higher H_2_S-to-thiosulfate ratio does not represent a secondary response but rather a compensatory effect to high BP load in the early stage of CKD children.

For further investigations of the impact of H_2_S against subclinical CVD in CKD children, we examined the correlation of H_2_S and thiosulfate with CVD risk markers, including LV mass, cIMT, and arterial stiffness parameters. Increases in LV mass and cIMT have been observed in adults and children with advanced CKD [15]. A lower plasma H_2_S level was reported as an independent predictor of increased LV mass in adult CKD [28]. This notion was supported by our data, which show a negative association between H_2_S and LV mass. We also found cIMT was positively associated with the H_2_S-to-thiosulfate ratio. In view of the fact that a high H_2_S-to-thiosulfate ratio represents increased conversion of thiosulfate to H_2_S, our data suggest an increase in this ratio might be a compensatory response against increased cIMT in children with early stages of CKD. Nevertheless, in the current study, we did not observe abnormal arterial stiffness parameters in children with early stages of CKD and their associations with H_2_S-related parameters. Given that validation studies of arterial stiffness have not been reproduced in childhood CKD [29], further studies are necessary to clarify the prognostic abilities of AI and PWV for cardiovascular outcomes in larger pediatric CKD cohorts.

Overall, the current study has some limitations. First, it is probable that a small number of CKD children from one hospital do not represent an entire population and provide sufficient power to determine slight differences in the CVD risk markers. Further multicenter studies of larger numbers may be warranted. Second, we did not recruit healthy children in this cohort since children with CKD stage G1 served as the control to compare the differences of BP load and CV risk markers between two different levels of eGFR (i.e., ≥90 vs. <90 mL/min/1.73 m^2^). Third, reference values for ABPM were based on studies conducted on participants of white ethnicity [20]. Ethnic differences must be considered, and our findings await further validation in other populations.

## 5. Conclusions

There is a paucity of data on the role of H_2_S on childhood CKD, especially in the early stage. Our study casts a new light on the different predictive abilities of plasma H_2_S, thiosulfate, and the H_2_S-to-thiosulfate ratio as biomarkers for subclinical CVD in children with CKD. A better understanding of the role of biomarkers will aid in risk stratification and personalized approaches for CKD care. Exploiting the possibilities that the study of H_2_S holds in this regard can have potential implications for therapeutic strategies against CVD in pediatric CKD.

## Figures and Tables

**Table 1:** Demographics and biochemical data.

CKD Stage	G1	G2–G4
Case numbers	35	21
Age (years)	10.7 (8.7–13.9)	14.4 (10.5–16.1) *
Male gender (%)	17 (42.9%)	15 (71.4%)
Body height (percentile)	50 (25–85)	50 (25–85)
Body weight (percentile)	75 (25–85)	75 (25–91)
Body mass index (kg/m^2^)	17.9 (15.1–20.9)	18.6 (15.6–23.1)
Systolic blood pressure (mmHg)	106 (100–112)	120 (112–125) *
Diastolic blood pressure (mmHg)	68 (63–77)	70 (64–80)
CAKUT (%)	15 (42.9%)	14 (66.7%)
Hypertension (% by office BP)	6 (20.7%)	6 (40%)
Blood urea nitrogen (mg/dL)	11 (10–13)	16 (12.5–18.5) *
Creatinine (mg/dL)	0.5 (0.45–0.55)	0.9 (0.75–0.96) *
eGFR (mL/min/1.73 m^2^)	120 (112–126)	80 (70–85) *
Hemoglobin (g/dL)	13.5 (13–14.1)	14.3 (12.9–15.5)
Total cholesterol (mg/dL)	158 (141–181)	155 (133–178)
Low-density lipoprotein (mg/dL)	80 (65–102)	75 (62.5–96.5)
Triglyceride (mg/dL)	61 (49–101)	65 (50.5–103)
Uric acid (mg/dL)	5.1 (4–6)	6.4 (5.7–7.7) *
Glucose (mg/dL)	87 (83–89.5)	86 (83–91.5)
Sodium (mEq/L)	141 (140–142)	141 (140–142)
Potassium (mEq/L)	4.3 (4.1–4.5)	4.5 (4.2–4.6)
Calcium (mg/dL)	9.5 (9.2–9.9)	9.8 (9.3–10.1)
Phosphate (mg/dL)	4.8 (4.5–5.2)	4.8 (4.2–5.1)
Urine total protein-to-creatinine ratio (mg/g)	52.5 (37.3–327.9)	43.3 (30.5–225.4)
On antihypertensive therapy	5 (14.3%)	6 (28.6%)

Values are presented as median (25th, 75th percentile) or *n* (%). * *p* < 0.05 by the Mann–Whitney U-test. eGFR = estimated glomerular filtration rate; CAKUT = congenital anomalies of kidney and urinary tract; CKD = Chronic Kidney Disease.

**Table 2:** Cardiovascular assessments.

CKD Stage	G1	G2–G4
24 h ABPM	35	21
Abnormal ABPM profile (with any of the following abnormalities)	22 (62.9%)	16 (76.2%)
Average 24 h BP > 95th percentile	4 (11.4%)	7 (33.3%) *
Average daytime BP > 95th percentile	2 (5.7%)	4 (19%)
Average nighttime BP > 95th percentile	9 (25.7%)	9 (42.9%)
BP load ≥ 25%	15 (42.9%)	10 (47.6%)
Nocturnal decrease in BP of <10%	21 (60%)	12 (57.1%)
Left ventricular mass (g)	86.4 (65.6–99.3)	118 (72.2–171) *
Left ventricular mass index (g/m^2.7^)	30.3 (25.9–36.1)	34.2 (28.4–42.2)
Carotid artery intima-media thickness (mm)	0.3 (0.3–0.4)	0.3 (0.3–0.4)
Augmentation index (%)	−2.8 (−10.2–0.8)	−6 (−16.8–−4.1)
Pulse wave velocity (m/s)	3.8 (3.4–4.1)	4 (3.6–4.7)

Values are presented as median (25th, 75th percentile) or *n* (%). * *p* < 0.05 by the Chi-squared test or the Mann–Whitney *U*-test. ABPM = 24 h ambulatory blood pressure monitoring. BP = blood pressure.

**Table 3:** Correlation between plasma H_2_S and thiosulfate levels with cardiovascular markers.

Cardiovascular Markers	H_2_S		Thiosulfate		H_2_S-to-Thiosulfate Ratio	
	*r*	*p*	*r*	*p*	*r*	*p*
24 h systolic blood pressure	0.239	0.076	−0.263	0.05	0.306	0.022 *
Daytime systolic blood pressure	0.234	0.082	−0.218	0.106	0.238	0.077
Nighttime systolic blood pressure	0.275	0.04 *	−0.27	0.044 *	0.336	0.011 *
24 h diastolic blood pressure	0.241	0.074	−0.124	0.362	0.18	0.184
Daytime diastolic blood pressure	0.139	0.307	−0.117	0.39	0.103	0.449
Nighttime diastolic blood pressure	0.226	0.094	−0.084	0.54	0.159	0.242
Left ventricular mass	−0.034	0.804	−0.093	0.495	0.095	0.484
Left ventricular mass index	−0.201	0.137	−0.129	0.343	0.045	0.742
Carotid artery intima-media thickness	0.244	0.07	−0.173	0.201	0.267	0.047 *
Augmentation index	−0.127	0.351	−0.115	0.397	0.076	0.579
Pulse wave velocity	0.004	0.979	−0.039	0.777	0.054	0.694

* *p* < 0.05 by Spearman’s correlation coefficient.

**Table 4:** Plasma H_2_S and thiosulfate levels vs. ABPM profile.

ABPM Profile	*n*	H_2_S	Thiosulfate	H_2_S-to-Thiosulfate Ratio
		μmol/L	μmol/L	μmol/μmol
24 h BP				
Normal	45	15.5 ± 9.6	1.07 ± 1.44	39.2 ± 46.9
Abnormal	11	18.1 ± 8.8	1.02 ± 1.47	82.3 ± 86.7 *
Daytime BP				
Normal	50	15.9 ± 9.8	1.05 ± 1.42	45.6 ± 59.1
Abnormal	6	17.1 ± 5.6	1.18 ± 1.65	64.7 ± 55.9
Nighttime BP				
Normal	38	14.3 ± 6.6	1.17 ± 1.54	30.3 ± 29.6
Abnormal	18	19.5 ± 13.1	0.84 ± 1.18	84.5 ± 84.1 *
BP load				
Normal	31	12.9 ± 5.3	1.1 ± 1.52	28.4 ± 28.8
Abnormal	25	19.8 ± 11.6 *	1.02 ± 1.35	71.7 ± 75.7 *
Night dipping				
Normal	23	13.9 ± 5.4	0.94 ± 1.05	30 ± 24.9
Abnormal	33	17.5 ± 11.3	1.15 ± 1.66	60 ± 71.3 *
ABPM profile				
Normal	18	13.1 ± 5.6	0.94 ± 1.11	24.7 ± 15
Abnormal	38	17.4 ± 10.6	1.12 ± 1.57	58.6 ± 67.9 *

* *p* < 0.05 by the *t*-test.

**Table 5:** Adjusted regression model estimates of the association of plasma H2S, thiosulfate, and their ratio with cardiovascular risk markers.

Dependent Variable	Explanatory Variable	Adjusted ^a^		Model	
		Beta	*p* Value	*r*	*p* Value
Nighttime SBP	H_2_S-to-thiosulfate ratio	0.356	0.009	0.488	0.002
24 h DBP	Thiosulfate	0.354	0.004	0.628	<0.001
Daytime DBP	Thiosulfate	0.317	0.012	0.589	<0.001
Nighttime DBP	Thiosulfate	0.32	0.012	0.574	<0.001
Left ventricular mass	H_2_S	−0.291	0.004	0.765	<0.001
cIMT	H_2_S-to-thiosulfate ratio	0.315	0.021	0.458	0.004

^a^ Adjusted for age, gender, eGFR, and uric acid. BP = blood pressure. SBP = systolic blood pressure. DBP = diastolic blood pressure. cIMT = carotid artery intima-media thickness.

## Data Availability

Data are contained within the article.

## Supplementary Materials

The following supporting information can be downloaded at: https://www.mdpi.com/article/10.3390/jpm12081241/s1, Table S1: Plasma H_2_S, thiosulfate, and H_2_S-to-thiosulfate ratio in CKD children.
