# Supplementary material for: Hydrogen Sulfide-to-Thiosulfate Ratio Associated with Blood Pressure Abnormalities in Pediatric CKD
Source: J Pers Med. 2022 Jul 29;12(8):1241. doi: 10.3390/jpm12081241 (PMC9409977; doi:10.3390/jpm12081241)
Supplement: Supplementary file 1 [file jpm-12-01241-s001.zip › Supplementary Table Tain.pdf]

**Table S1.** Plasma $H_2S$, thiosulfate and $H_2S$-to-thiosulfate ratio in CKD children.

| | | $H_2S$ μM/L | Thiosulfate μM/L | HTR μM/μM |
|---|---|---|---|---|
| CKD Stage | n | | | |
| Stage 1 | 35 | 16.8 ± 10.5 | 1.18 ± 1.6 | 49.2 ± 67.5 |
| Stage 2 | 17 | 14.3 ± 5.3 | 0.9 ± 1.21 | 44.4 ± 42.1 |
| Stage 3 | 3 | 8.8 ± 1.04 | 0.76 ± 0.9 | 39.1 ± 45.7 |
| Stage 4 | 1 | 37.8 | 0.51 | 74.2 |

Data are mean ± SD or actual number. HTR = $H_2S$-to-thiosulfate ratio.
